# Toxic leukoencephalopathy with axonal spheroids caused by chemotherapeutic drugs other than methotrexate

**DOI:** 10.1186/s12883-022-02818-8

**Published:** 2022-08-03

**Authors:** Ka Young Lim, Seong-Ik Kim, Hyunhee Kim, Jeongwan Kang, Jin Woo Park, Jae Kyung Won, Dong-Yeop Shin, Sung-Hye Park

**Affiliations:** 1 Department of Pathology, Seoul National University College of Medicine, 103 Daehak-ro, Jongno-gu, Seoul, 03080 Republic of Korea; 2 Internal Medicine, Seoul National University College of Medicine, Seoul, 03080 Republic of Korea; 3 Institute of Neuroscience, Seoul National University College of Medicine, Seoul, 03080 Republic of Korea

**Keywords:** Autopsy, Chemotherapy, Chemobrain, Leukoencephalopathy, Axonal spheroids

## Abstract

**Background:**

The objective of this report is to share the clinicopathological features of chemotherapy-induced toxic leukoencephalopathy, which is a rare and under-recognized disease, clinically characterized by rapidly progressive cognitive loss that often leads to sudden death.

**Case presentation:**

A 64-year-old woman and a 63-year-old man, who had both suffered from a rapid deterioration of consciousness, were autopsied under the clinical impressions of either the central nervous system graft versus host disease (CNS-GVHD), infectious encephalitis, or autoimmune encephalitis. Both patients had been treated with multiple chemotherapy regimens, including adriamycin, cytarabine arabinoside, daunorubicin, fludarabine, azacitidine, and allogeneic peripheral blood stem cell transplantation to treat hematological malignancies (acute myelogenous leukemia and myelodysplastic syndrome). Neuropathological findings at autopsy revealed rarefaction and vacuolar changes of the white matter with axonal spheroids, reactive gliosis, and foamy macrophage infiltration, predominantly in the visual pathways of the occipital and temporal lobes. Damaged axons exhibited immunoreactivity to beta-amyloid, consistent with axonopathy. However, there was no lymphocyte infiltration that suggested CNS-GVHD or any type of encephalitis.

**Conclusion:**

The neuropathology found in the presented cases had the characteristic features of toxic leukoencephalopathy (chemobrain). Our cases showed that toxic leukoencephalopathy can also be caused by chemotherapy drugs other than methotrexate.

## Background

Chemotherapy-induced toxic (leuko-) encephalopathy, or “chemobrain,” is clinically characterized by progressive cognitive disorders that often result in sudden death [1, 2]. Chemotherapy-related cognitive dysfunction has become an increasing concern because long-term cancer survivors are increasing dramatically. However, the exact incidence is unknown.

In 1972, Kay et al*.* first reported eight patients with acute leukemia who developed methotrexate treatment-related encephalopathy, and found necrosis of blood vessel walls upon autopsy [3, 4]. In 1975, Rubinstein et al*.* described disseminated necrotizing leukoencephalopathy (DNL) after chemotherapy in children with acute lymphoblastic lymphoma (ALL) and Burkitt’s lymphoma [5]. The patients had clinically progressive dementia, coma, death, and pathologically diagnosed DNL. In 2009, Matsubayashi et al*.* introduced a new type of chemotherapy-induced disseminated demyelinating leukoencephalopathy (DDL), distributed in the occipital lobe and characterized by extensive demyelination without significant axonal changes [6].

In 2011, Uwe Schlegel emphasized that neurological complications of chemotherapy should be separated from central nervous system (CNS) dysfunctions caused by the tumor itself [7]. He defined chemotherapy-induced neurotoxic complications, listed their respective causative agents, and classified clinical syndromes caused by cytostatic drugs into 11 categories: acute encephalopathy, subacute encephalopathy, chronic encephalopathy, posterior reversible (leuko-) encephalopathy syndrome (PRES), multifocal leukoencephalopathy, thrombotic microangiopathy, cerebral infarctions, cortical blindness, cerebellar dysfunction, seizure, and aseptic meningitis. PRES is clinically characterized by headaches, visual disturbances, confusion, seizures, and eventually coma. And although not a part of the 11 categories, myelopathy can be a complication of methotrexate and cytarabine.

The definition of leukoencephalopathy is a structural alteration of cerebral white matter, mostly myelination disorders, but any type of white matter disease [8]. Here, we report two cases of autopsy-proven toxic leukoencephalopathy caused by various chemotherapy regimens other than methotrexate. This study highlights the unique clinicopathological features that can help clinicians avoid misdiagnosing patients with similar cases.

## Methods

The primary clinical information and autopsy findings were reviewed by two pathologists (SHP and JKW). The brain-only autopsies were conducted on two deceased patients with routine methods at the Seoul National University Hospital (SNUH) Brain Bank. Hematoxylin and eosin (H&E) Luxol fast blue (LFB), Gomori’s methenamine silver (GMS), and Periodic acid Schiff stains (PAS) staining and immunohistochemical analysis were carried out on formalin-fixed, paraffin-embedded (FFPE) blocks. Primary antibodies (Table 1) for immunohistochemistry included NeuN (1: 100, DAKO, Glostrup, Denmark), synaptophysin (1: 200, DAKO), GFAP (1: 300, DAKO), neurofilament (NF, 1: 200, DAKO), CD3 (1: 200, DAKO), CD8 (1: 200, DAKO), CD20 (1: 200, DAKO), CD68 (1: 200, DAKO), and TMEM119 (1: 200, DAKO). Luxol fast blue (LFB) and myelin basic protein (MBP, 1: 200, Cell Marque, Rocklin, US) stains were carried out to determine the demyelination status. To rule out neurodegenerative disorders, immunohistochemical stains of β-amyloid (1: 200, DAKO), phosphorylated tau (AT8) (1: 200, DAKO), α-synuclein (1: 200, DAKO), and TDP43 (1: 200, DAKO) were analyzed. Positive antibody controls were brain tissues from patients with Alzheimer's disease (especially for pTau and beta-amyloid), Parkinson's disease (especially for α-synuclein), and limbic-predominant age-related TDP43 encephalopathy (especially for TDP43). As a negative control, the primary antibodies were omitted during immunostaining.

**Table 1:** Primary antibodies used in our two autopsy cases for diagnosis

Antibodies	Dilution	Company	Findings in Case 1 and Case 2
GFAP	1:300	DAKO, Glostrup, Denmark	+ in the reactive astrocytes
NeuN	1: 500	Millipore, Temecula, USA	+ in the neurons
Neurofilament (NF)	1: 2000	DAKO, Glostrup, Denmark	+ in the axons and axonal spheroids
Phosphorylated NF	1: 10,000	Millipore, Temecula, USA	+ in the axons and axonal spheroids
Synaptophysin	1: 100	Novocastra, Newcastle, UK	+ in the gray matter
TMEM	1: 500	ABCAM, Bristol, UK	+ in the intrinsic microglia
CD163	1: 200	ABCAM, Bristol, UK	+ in the pigmented microglia
CD68	1: 2000	DAKO, Glostrup, Denmark	+ in the exogenous macrophages
CD3	1: 100	DAKO, Glostrup, Denmark	Negative in the entire brain
CD20	1: 100	DAKO, Glostrup, Denmark	Negative in the entire brain
α-synuclein	1: 200	ABCAM, Bristol, UK	Negative in the entire brain
β-amyloid	1: 500	Covance, Dallas, USA	Negative in the entire brain
3 repeat (3R) tau	1: 100	Millipore, Ontario, Canada	Negative in the entire brain
4 repeat (4R) tau	1: 1000	Millipore, Ontario, Canada	Negative in the entire brain
p-Tau (AT8)	1: 100	ThermoFisher, Waltham, USA,	Negative in the entire brain
p-TDP43	1: 1,000	Cosmobio, Tokyo, Japan	Negative in the entire brain

*GFAP* Glial fibrillary acidic protein, *NeuN* Neuronal nuclei, *CD* Cluster of differentiation, *p-Tau* phosphorylated-Tau, *p-TDP43* Phosphorylated TAR DNA binding protein 43

## Case presentation

### Case 1

**Fig. 1:**
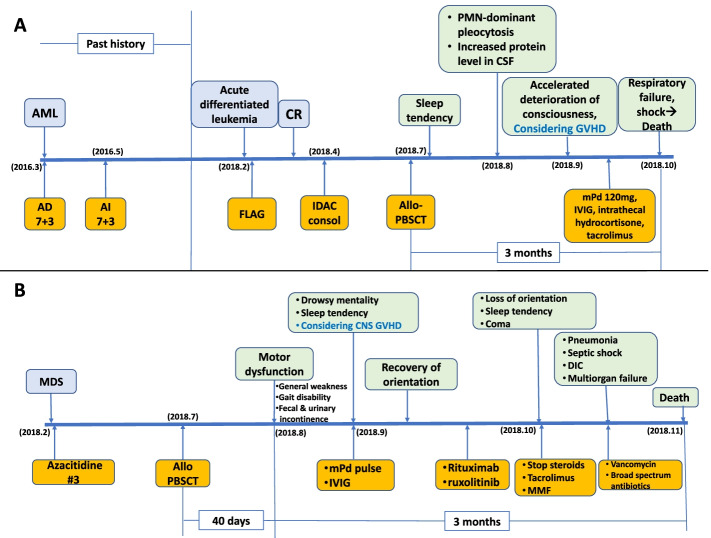
**A** Patient 1 was diagnosed with AML in 2016 and treated with standard induction chemotherapy of cytarabine arabinoside, and daunorubicin (AD 7 + 3), and further re-induction chemotherapy of cytarabine and idarubicin (AI 7 + 3). No consolidation chemotherapy was performed due to the patient’s refusal. Unfortunately, the patient’s disease relapsed as acute undifferentiated leukemia. She was treated with salvage chemotherapy of fludarabine, and cytosine arabinoside, and G-CSF (FLAG) and achieved complete remission (CR). She was treated with one cycle of intermediate-dose Ara-C consolidation (IDAC). Then she underwent an allogeneic peripheral blood stem cell transplant (Allo-PBSCT). After approximately one month, she had a tendency to sleep and deterioration of consciousness was accelerated. Polymorphonuclear (PMN) leukocyte dominant pleocytosis had developed. Under the clinical impression of CNS-GVHD, methylprednisolone (mPD, 120 mg), intravenous immunoglobulin (IVIG), and intrathecal hydrocortisone therapy were implemented. However, she showed drowsy mentality acutely and died of respiratory failure. It was only three months after she began to develop a tendency to sleep. **B** Patient 2 was diagnosed with myelodysplastic syndrome (MDS) and was treated with three cycles of azacytidine for 3 months. He received allogeneic peripheral blood stem cell transplant (Allo PBSCT). Approximately one month later, he developed motor dysfunction (general weakness, gait disability, and fecal and urinary incontinence). Then sleep tendency and disorientation developed. Under the clinical impression of CNS-GVHD, methylprednisolone (mPD) pulse plus IVIG, rituximab and ruxolitinib were implemented. These symptoms improved temporarily but got worse. He was given tacrolimus and mycophenolate mofetil (MMF) instead of mPd pulse and IVIG. However, the patient’s condition deteriorated rapidly, and within three months after dyskinesia, he died of pneumonia, septic shock, disseminated intravascular coagulopathy (DIC), and multiorgan failure

**Fig. 2:**
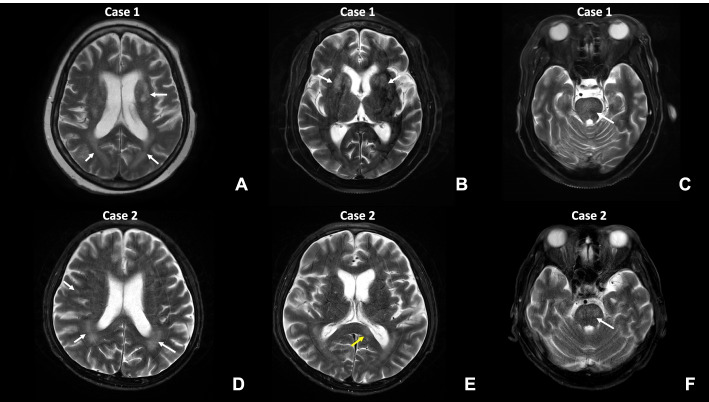
**A**, **B**, **C** T2-weighted brain MRI of case 1 revealed multiple high signal intensity lesions (arrows) in the white matter with diffuse high signal intensity of the optic pathway and central pons (arrow). **D**, **E**, **F** T2-weighted image of case 2 showed high signal intensity lesions (arrows) in the white matter, the splenium of the corpus callosum (yellow arrow) and the pons (arrows)

A 64-year-old woman with acute myeloid leukemia received chemotherapy, first with adriamycin (AD), cytarabine arabinoside (cytarabine), and daunorubicin (AD 7 + 3), then with AD, cytarabine, and idarubicin (AI 7 + 3) (Fig. 1, Table 2). No consolidation chemotherapy was performed, because the patient had refused. Unfortunately, the disease relapsed as acute undifferentiated leukemia. She was treated with salvage chemotherapy of fludarabine, cytosine arabinoside, and granulocyte-colony stimulating factor (G-CSF) (FLAG) and achieved complete remission (CR). She was treated with one cycle of intermediate-dose Ara-C consolidation (IDAC). Then, she underwent a full match allogeneic peripheral blood stem cell transplant (Allo-PBSCT) from a male donor, registered in the Korea Marrow Donor Program. After approximately one month, she had a tendency to sleep and the deterioration of consciousness accelerated. Polymorphonuclear (PMN) leukocyte dominant pleocytosis had developed. Cerebrospinal fluid (CSF) examination revealed polymorphonuclear neutrophil (PMN)-dominant pleocytosis and increased protein level up to 135 g/dL (cf. normal range: 15–45 g/dL). Three months after transplantation, the patient’s consciousness deteriorated rapidly, but light reflexes were still intact. However, she could neither respond to painful stimuli nor communicate. Electroencephalography revealed generalized slow activity indicating diffuse cerebral dysfunction. T2-weighted magnetic resonance imaging (MRI) showed high signal intensity in the bilateral centrum semiovale and the optic pathway with a slightly decreased size of bilateral basal ganglia (Fig. 2). Graft versus host disease (GVHD), toxic encephalopathy, metabolic encephalopathy, and autoimmune encephalitis were considered as the clinical differential diagnosis. The patient was treated with methylprednisolone (120 mg), intravenous immunoglobin (IVIG) (400 mg/kg) and intrathecal hydrocortisone under the assumption of CNS-GVHD. Autoimmune encephalitis could be ruled out by neurologists upon getting negative results of anti-aquaporin4 IgG Ab, anti-monosialoganglioside (GM1) Ab, and anti-ganglioside Ab IgG panel (anti-GQ1b IgM, anti-GD1 B IgM) tests from peripheral blood serum samples.

**Table 2:** Clinicopathological findings of two cases with autopsy-proven chemobrains

	Case 1	Case 2
Age/Gender	64/F	63/M
Active problem	Loss of consciousness, sleep tendency, type I respiratory failure due to aspiration pneumonia and sepsis	Disorientation, drowsy mentality, sleep tendency, coma Pneumonia, sepsis, & DIC, multiorgan failure
Underlying disease	Relapsed Acute undifferentiated myelogenous leukemia	Myelodysplastic syndrome excess blast 1 (MDS-EB1)
Chemotherapy-Regimen	FLAG, IDAC	Azacitidine #3
Transplant	MUD-alloPBSCT	MUD-alloPBSCT
Autopsy finding	Numerous axonal spheroid with vacuolar changes of the white matter and exogenous foamy macrophage (CD68-positive/TMEM119-negative) infiltration Beta-amyloid deposit in the degenerated axons of the optic tract of occipital and temporal lobes	Numerous axonal spheroids with vacuolation of the white matter with exogenous foamy macrophage (CD68-positive/TMEM119-negative) infiltration Beta-amyloid deposit in the axons, extensively in the most white matter
Main involving area	The white matter of the occipital and temporal lobe	The white matter of the occipital, temporal, and parietal lobe
T- or B-lymphocytes	Absent in the entire brain parenchyma	Absent in the entire brain parenchyma
Survival	3 months after alloPBSCT	4 months after alloPBSCT (about 3 months after onset of disorientation)

*FLAG* (fludarabine, Ara-Cytarabine, granulocyte colony-stimulating factor), *IDAC* Intermediate-dose Ara-Cytarabine, *MUD* Matched unrelated donor, *allPBSCT* Allogeneic peripheral blood stem cell transplantation

In addition to diffuse cerebral dysfunction, the patient developed respiratory failure and shock, which appeared to be caused by sepsis and aspiration pneumonia, despite the aggressive use of broad-spectrum antibiotics. X/Y fluorescence in situ hybridization of the peripheral blood revealed 0.2% of the XX chromosome of the host and 99.8% of the XY chromosome of the male blood donor. The short tandem repeat examination showed her remnant host blood cells were 5.6%. The patient’s condition rapidly deteriorated and she soon passed away. An autopsy was performed to determine brain pathology.

**Fig. 3:**
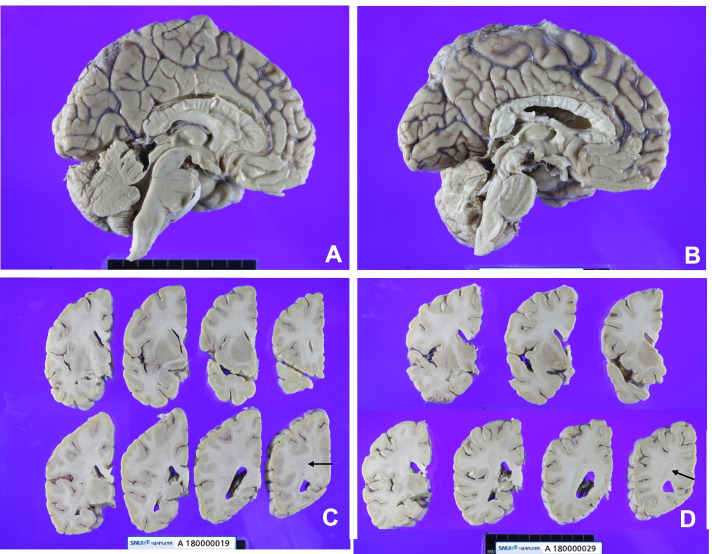
**A**-**D** Gross histology of both cases revealed mild global atrophy. The brain in case 1 weighed 1060 g and weighed 1310 g in case 2. The cut surface of the brain showed irregular discoloration of the white matter (arrows) (**A**, **C**: Case 1, **B**, **D**: Case 2)

The brain at autopsy revealed symmetric global atrophy, pale parenchyma, and pale substantia nigra (Fig. 3). The brain weighed 1060 g, and the CSF was clear. Atherosclerosis was found in a short segment of the basilar artery. On microscopic exam, atrophy of the globus pallidus with marked neuronal loss and reactive gliosis were found, as well as an indistinguishable globus pallidus interna and externa in the basal ganglia.

**Fig. 4:**
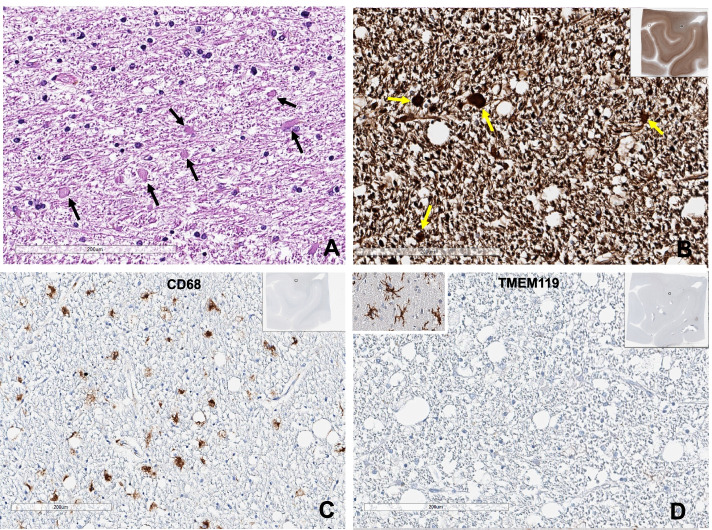
Microscopic findings of Case 1. **A** Hematoxylin and eosin stain of the optic pathway of the left occipital lobe in case 1 revealed many axonal spheroids (arrows). **B** Neurofilaments (NF) stain revealed several oval-to-round axonal spheroids (yellow arrows) with the vacuolar change of the optic pathway of the left occipital lobe. **C** CD68 stain revealed many CD68-positive foamy macrophages in the optic pathway of the left occipital lobe. **D** TMEM119 stain was negative, suggesting complete loss of microglia in the optic pathway of the occipital lobe. The left inlet show TMEM-positive microglia in the neocortex of this patient (Bar A-D: 200 µm)

Spongiform (vacuolar) changes of the white matter, axonal spheroids, foamy macrophage infiltration, and reactive gliosis in the white matter, especially in the optic pathway of the occipital and temporal lobes, are all consistent with previously reported chemotherapy-induced brain changes (Fig. 4). CD68-positive and TMEM119-negative foamy macrophages infiltrated the cerebral white matter and the optic pathway of the occipital and temporal lobes (Fig. 4). Also, TMEM119-positive microglia were markedly reduced in the white matter of the occipital lobe, but preserved in the gray matter.

**Fig. 5:**
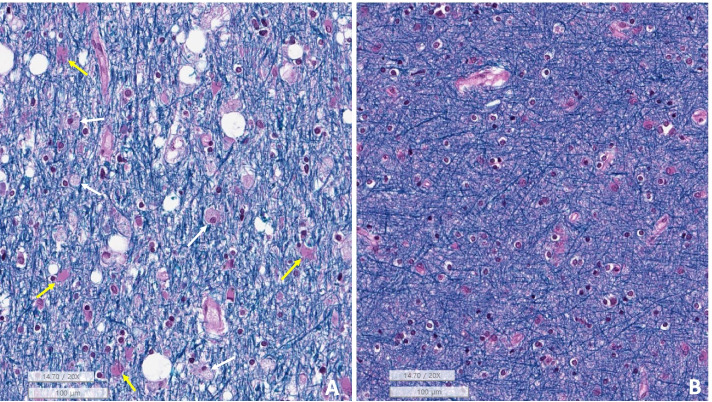
**A** Luxol fast blue (LFB) stain of case 1 showed rarefaction of myelinated nerve fibers with vacuolar change of the white matter compared to **B** the non-involving areas of this case. There were scattered reactive astrocytes (arrows) (**A**, **B**: LFB stain, underbar scale: 100 µm)

White matter rarefaction was identified in the centrum semiovale with LFB (Fig. 5) and MBP stains. Many β-amyloid-positive diffuse-type senile plaques were found in the neocortex, putamen, subiculum, and entorhinal cortex (Fig. 7A), but no neuritic plaques were present [Thal phase 2 and consortium to establish a registry for Alzheimer’s disease (CERAD) stage 0].

The globus pallidus showed a severe neuronal loss and reactive gliosis. Although a focal loss of dopaminergic neurons was identified in the midbrain, there was no Lewy body in the brain, including the olfactory bulb and the brainstem. AT8, α-synuclein, and p-TDP43 were completely negative in the brain tissue, suggesting no other neurodegenerative disorders except a primary age-related tauopathy (PART) which was confirmed with AT8 immunohistochemistry in the entorhinal cortex. There was no evidence of cerebral amyloid angiopathy. Iron stain showed no iron deposits throughout the brain, including the basal ganglia. GMS and PAS stains revealed no fungal organisms. There was no evidence of viral or autoimmune encephalopathy. The possibility of a donor-derived inflammatory cell infiltration was carefully investigated, but given that there was neither any inflammatory cell infiltration on H&E stain, nor CD3, CD8, and CD20 immunostaining in the whole brain, CNS-GVHD could be ruled out. The final diagnosis was chemotherapy-induced toxic leukoencephalopathy with predominant involvement of optic pathway suggesting PRES.

### Case 2

A 63-year-old male patient with an underlying alcoholic liver cirrhosis and histories of multiple transfusions due to anemia diagnosed with myelodysplastic syndrome (MDS). He was treated with bridging azacytidine #3 for MDS for three months and received busulfan-fludarabine-anti-thymocyte globulin (BuFluATG) conditioning (Fig. 1). He then received a full match hematopoietic stem cell transplant from an unrelated male donor, registered in the Korea Marrow Donor Program. One month after transplantation, the patient began displaying general weakness, gait disability, and combined fecal and urinary incontinence. To prevent CNS-GVHD, tacrolimus was administered for 13 days. Forty days after transplantation, the patient became drowsy and rapidly lost consciousness. Fifty days after transplantation, he was unable to maintain eye contact and had a cognitive disorder with a sleep tendency. Electroencephalography revealed generalized slow activity with intermittent rhythmic delta slow waves, maximal in both frontotemporal regions, showing diffuse cerebral dysfunction with nonconvulsive epileptic conditions. Brain MRI revealed a restrictive lesion on the left splenium of the corpus callosum, multiple T2 high signal intensity lesions at the central pons, and bilateral parietal periventricular white matter (Fig. 2). Based on the MRI findings and the increased CSF protein levels (81 g/dL), the differential diagnoses of CNS-GVHD, CNS infection, or autoimmune encephalitis were suggested by neurologists. However, anti-aquaporin4 IgG Ab, anti-monosialoganglioside (GM1) Ab, and anti-ganglioside Ab IgG panel (anti-GQ1b IgM, anti-GD1 B IgM) tests from peripheral blood serum samples were all negative.

After administration of steroids (methylprednisolone 1 g/day for 5 days), IVIG (400 mg/kg for 5 days) and levetiracetam [500 mg bid (twice a day) IV], the patient recovered his ability to speak his name. However, he soon fell into a coma. Under suspicions of CNS-GVHD, tacrolimus IV 0.04 mg/kg/day was stated with a targeting dose of 10–20 ng/mL. Methylprednisolone and IVIG were then administered, following which the patient briefly recovered orientation. Rituximab and ruxolitinib were administered to suppress immune reactions. However, the patient once again lost orientation, showed sleep tendency, and fell back into a comatose status. Steroids were stopped, and tacrolimus and mycophenolate mofetil (MMF) were administered, but the patient’s consciousness deteriorated, breathing tones becoming coarse. Blood culture tested positive for extended-spectrum beta-lactamases (ESBL)-positive Klebsiella. He was diagnosed with ESBL-positive Klebsiella pneumonia and septic shock with disseminated intravascular coagulopathy. Although vancomycin was added to the broad-spectrum antibiotic therapy, he succumbed to multiorgan failure four months after transplantation.

**Fig. 6:**
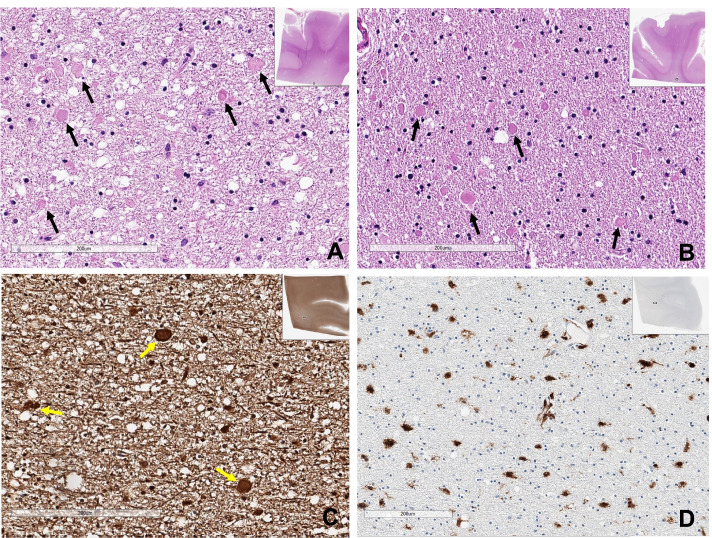
Microscopic findings of Case 2. **A** The left inferior parietal lobe and **B** the left inferior temporal lobe had many variable-sized axonal spheroids (arrows) with the vacuolar change of the white matter. **C** The optic pathway of the left occipital lobe showed oval shape neurofilaments-positive axonal spheroids (arrows). **D** The white matter of the left motor cortex showed scattered CD68-positive large macrophages (Bar **A**-**D**: 200 µm)

**Fig. 7:**
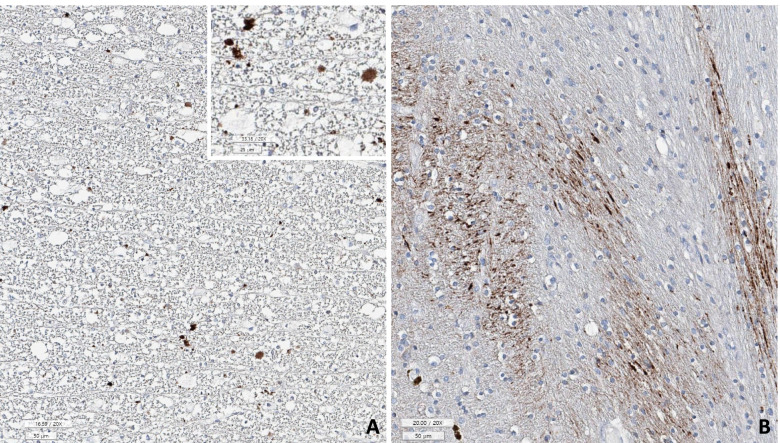
**A** Beta-amyloid stain showed amyloid deposit in the axons of the involving white matter (case 1). **B** Axonal beta-amyloid deposit was severe in the case 2, suggesting axonal damage (**A**, **B**: beta-amyloid immunostain, underbar scale: 50 µm)

The brain at autopsy showed no gross abnormalities, and the brain weighed 1310 g (Fig. 3). Atherosclerosis and arteriosclerosis were not identified. However, H&E, GFAP, NF, LFB, CD68, and TMEM119 stains revealed white matter vacuolization with diffuse axonal spheroids, reactive gliosis, and foamy macrophage infiltration in the cerebral white matter and the optic pathway of the temporal and occipital lobes (Fig. 6). These findings were identical to that of case 1. Beta-amyloid stain was deposited in the axons of the white matter of the brain, suggesting axonal injury, which was more severe in case 2 than in case 1 (Fig. 7B). GFAP exhibited reactive gliosis of the entire neocortex. Many pTau (AT8)-positive neurofibrillary tangles and tau-positive neuropil threads (Braak stage II) were found in CA1 and CA2 of the hippocampus and entorhinal and transentorhinal cortex, consistent with PART, but there was no evidence of other neurodegenerative disorders when analyzed immunohistochemically with AT8, α-Synuclein, β-amyloid, and TDP43. Similar to case 1, CD3, CD8, and CD20 immunostaining showed no lymphocytic infiltration in the whole brain, which ruled out the diagnosis of GVHD. The final autopsy diagnosis was chemotherapy-induced toxic leukoencephalopathy with a predominant optic pathway involvement compatible with PRES, but the patient accumulated irreversible damage in the brain and eventually passed away.

## Discussion

Systemic chemotherapy, often using a combination of multiple drugs, is the treatment of choice for patients with malignancies, particularly hematopoietic cell malignancies. “Chemotherapy-induced toxic (leuko-) encephalopathy” is a rising clinical syndrome characterized by a progressive cognitive disorder that severely lowers the quality of life and can even lead to sudden death. However, few cases are known, most of which are methotrexate-induced leukoencephalopathy [3, 6, 9, 10].

Our two cases of autopsy-proven toxic leukoencephalopathy caused by chemotherapy other than methotrexate shared some unique clinicopathologic findings. Both patients showed cognitive impairment and neurological deficit, such as motor and sensory loss, after multiple courses of various chemotherapy including fludarabine. They also progressed rapidly to coma and death.

The development of mental changes and the rapid progression of neurological deterioration after allogeneic PBSCT from matched unrelated donors were clinically suggestive of CNS-GVHD or autoimmune encephalitis. However, no autoantibodies were found in patients’ blood. Unfortunately, we did not test for autoantibodies in the CSF. If autoimmune or paraneoplastic encephalitis is clinically suspected, autoantibodies against neuronal cell-surface or synaptic proteins should be evaluated [11]. Chemotherapy-related toxic leukoencephalopathy is characterized by white matter changes, including axonal spheroids, white matter vacuolization, macrophage infiltration, edema, and reactive gliosis, along with long tract degeneration [12]. In the presenting cases, brain autopsies revealed all the features of above mentioned toxic leukoencephalopathy, including spongiform changes in the cerebral white matter and axonal shperoids. Beta-amyloid deposit in the axons suggested axonal injury. CD3, CD8, and CD20 stains did not reveal lymphocytic infiltration in both cases, ruling out CNS-GVHD. Apoptotic cells were not identified. The pathological findings of these two cases were consistent with chemotherapy-induced toxic leukoencephalopathy [1, 2, 9, 12–15]. Pavletic et al*.* reported on drug-induced parkinsonism after allogeneic bone marrow transplantation [16], but there was no evidence of Parkinson's disease in either of our cases, both clinically and pathologically. There were no Lewy bodies or alpha-synuclein deposits in the brains.

Although white matter diseases with numerous axonal spheroids mimic the pathology of colony stimulating factor-1 receptor (CSF-1R)-mutant leukoencephalopathy, such as dentatorubral-pallidoluysian atrophy (DRPLA) [17]. However, in our cases, there were no progressive myoclonus, epilepsy, ataxia, or dementia, which are the typical symptoms of DRPLA. DRPLA is an autosomal dominant inherited disease, but our two cases do not have such a family history. Pathologically, the axonal spheroids were restricted to the occipital and temporal lobes and parietal lobe where the optic nerve pathway was mainly involved without pigmented macrophages. Pigmented macrophages are usually present in DRPLA.

Methotrexate is the most common drug associated with chemobrain, characterized by acute (reversible) encephalopathy, subacute encephalopathy, chronic encephalopathy, cerebral infarctions, seizure, or aseptic meningitis [6]. However, other chemotherapy drugs have also been associated with symptoms of toxic leukoencephalopathy that present with the symptoms mentioned above. Chronic encephalopathy and PRES have been related to high-dose multidrug chemotherapies, including cyclophosphamide, Cytarabine, cis-platinum, ifosfamide, vincristine, gemcitabine, and other immunosuppressants [18]. Chronic encephalopathy usually develops after a latency of some months to years, often presenting with progressive and irreversible clinical manifestations. PRES is clinically characterized by headaches, visual disturbances, confusion, seizures, and eventually coma.

In 2004, Lai et al*.* reported an autopsy-proven methotrexate-based chemotherapy-induced leukoencephalopathy in primary CNS lymphoma [19]. Unfortunately, treatment-related leukoencephalopathy is the leading brain pathology after the successful treatment of primary CNS lymphoma (PCNSL). Lai et al*.* reviewed five more autopsied patients who died of leukoencephalopathy [19] and concluded that chemotherapy-induced toxic leukoencephalopathy can develop very early after chemoradiation and is not always so delayed. They also suggested that vascular disease may be a component of this injury [19].

The chemobrain in our cases might be due to fludarabine, cytarabine, or busulfan, which are known to cause neurological side effects. High-dose fludarabine may cause cortical blindness, which has been associated with the administration of immunosuppressive drugs, antibodies, and other substances [12]. Cytarabine can induce cerebellar dysfunction and aseptic meningitis [14, 19]. Busulfan, cyclosporine, vincristine, cis-platinum, methotrexate, and paclitaxel may cause seizures [12]. The frequency and severity of CNS toxicity depend on the type of drug, cumulative doses, the duration of treatment, and additional risk factors such as coexisting neurological morbidity [20]. Well-known factors that increase the risk are dose escalation, combination therapy, stem cell transplantation, and irradiation of the brain [14].

In 1994, Cheson et al*.* reviewed the neurotoxicity of widely used purine analogs for indolent lymphoid malignancies, including fludarabine, cladribine, and pentostatin [21]. All three drugs cause life-threatening or fatal neurotoxicity at higher-than-recommended doses. Neurological complications with each drug occur in approximately 15% of patients at the recommended dose and are mostly mild and reversible. However, it can sometimes lead to severe neurological deficits that are delayed, sometimes partially reversible, other times fatal. [21].

In 1994, Zabernigg reported on the late-onset fatal neurotoxicity induced by low-dose fludarabine monotherapy in patients with B-cell chronic lymphocytic leukemia [15]. A 55-year-old man developed a severe neurological disorder, such as aphasia, apraxia, acalculia, hemihypesthesia, and spastic hemiparesis, six months after completing six cycles of fludarabine monotherapy. The pathological diagnosis was progressive multifocal leukoencephalopathy. He was in a coma until his death [15]. Although low doses of fludarabine were used, the spectrum and severity of neurotoxicity did not differ from that of the high doses.

The underlying cellular mechanisms of chemobrain are still unclear. However, some models based on clinical and animal experiments help us to speculate on the possible mechanisms. First, peripheral cytokines initiate the development of the chemobrain [22–24]. This cytokine-mediated signaling cascade induces persistent epigenetic alterations. These epigenetic changes alter gene expression, metabolic activity, and neuronal transmission that ultimately affect cognitive function. Chemotherapy drugs cause cellular stress and injury [22–24]. Axonal spheroids and axonal beta-amyloid deposits suggested axonal injury because beta-amyloid is a known marker for axonal injury [25]. This induces an inflammatory response in the periphery, releasing cytokines such as tumor necrosis factor-α (TNF-α), interleukin-6 (IL-6), IL-8, IL-10, and monocyte chemoattractant protein-1 (MCP-1). Peripheral cytokines can access the brain through leaky lesions in the blood–brain-barrier or can be transported via active transport [26, 27].

It is assumed that peripheral cytokines communicate with the cytokines of the CNS through the local inflammatory network [28]. Peripheral cytokines stimulate endothelial cells and perivascular macrophages, monocytes, and T cells in the brain to produce similar local cytokines and chemokines [26]. Microglia, astrocytes, oligodendrocytes, and neurons respond by releasing additional cytokines and chemokines in the brain. As a consequence of this cascade, oxidative stress increases, neurogenesis, and neuroplasticity decrease, and neuronal excitotoxicity increases [23]. These factors ultimately influence neurotransmitters and neuronal alteration [10, 23, 29]. Cytokines in the CNS are known to be mainly derived from microglia [23, 27]. Along with the strong evidence correlating peripheral cytokines and cognitive dysfunction, microglial activation has been identified as a key factor in the reactivation of astrocytes and dysfunctional oligodendrocyte precursor cells in a previous work [30]. TMEM119 is a reliable microglial marker, as it distinguishes microglia from circulating macrophages that flow into the brain [31].

 A timely report by Han et al. shows inhibition of CSF-1R as a potential therapeutic strategy for white matter disorders in the cases of neurodegenerative diseases [32]. However, it is uncertain whether CSF-IR inhibitors can act as a prophylactic agent in toxic leukoencephalopathy such as chemobrain, since they only reduce the number of microglia. In our cases, TMEM119-positive microglia decreased, while exogenous macrophages (CD68-positive and TMEM-negative) increased in the white matter. Tipton et al*.* also reported that presymptomatic immunosuppression with steroids could prevent CSF-1R-related leukoencephalopathy [33]. But steroids were ineffective in our cases, probably because it was implemented late after symptom onset. As mentioned above, there was no evidence of CSF-1R-related leukoencephalopathy in our cases.

## Conclusion

Autopsies of two patients revealed white matter diseases. Visual pathway in the occipital and temporal lobes had vacuolar changes in the white matter, with many axonal spheroids, beta-amyloid positive axons, and reactive gliosis. These white matter pathologies are the hallmarks of chemotherapy-related toxic leukoencephalopathy. Our cases show that toxic leukoencephalopathy can be caused by chemotherapy agents other than methotrexate. Without autopsy, no suspicion would have been raised, and the cause of death would have remained a mystery. The autopsy can exclude the diagnoses of autoimmune encephalitis, infectious encephalopathy, and CNS-GVHD.

## Data Availability

The datasets used during the current study are available from the corresponding author on request. The identifying/confidential patient data should not be shared.
